# Improving the peer-review process and editorial quality: key errors escaping the review and editorial process in top scientific journals

**DOI:** 10.7717/peerj.1670

**Published:** 2016-02-09

**Authors:** Antoni Margalida, Mª Àngels Colomer

**Affiliations:** 1 Department of Animal Science (Wildife Division)—Faculty of Life Sciences and Engineering, University of Lleida, Lleida, Spain; 2 Division of Conservation Biology. Institute of Ecology and Evolution, University of Bern, Bern, Switzerland; 3 Department of Mathematics—Faculty of Life Sciences and Engineering, University of Lleida, Lleida, Spain

**Keywords:** Bibliometric analyses, Corrections, Publishing, Mistake index, Peer review

## Abstract

We apply a novel mistake index to assess trends in the proportion of corrections published between 1993 and 2014 in *Nature*, *Science* and PNAS. The index revealed a progressive increase in the proportion of corrections published in these three high-quality journals. The index appears to be independent of the journal impact factor or the number of items published, as suggested by a comparative analyses among 16 top scientific journals of different impact factors and disciplines. A more detailed analysis suggests that the trend in the time-to-correction increased significantly over time and also differed among journals (*Nature* 233 days; *Science* 136 days; PNAS 232 days). A detailed review of 1,428 errors showed that 60% of corrections were related to figures, authors, references or results. According to the three categories established, 34.7% of the corrections were considered *mild*, 47.7% *moderate* and 17.6% *severe,* also differing among journals. Errors occurring during the printing process were responsible for 5% of corrections in *Nature*, 3% in *Science* and 18% in PNAS. The measurement of the temporal trends in the quality of scientific manuscripts can assist editors and reviewers in identifying the most common mistakes, increasing the rigor of peer-review and improving the quality of published scientific manuscripts.

## Introduction

Measuring the scientific quality of journals in regards to the peer-review and editing process is important to improving the standards and rigor of the scientific literature. Several indicators of the health of scientific production have been used such as the frequency of retracted publications (Fang, Grant Steen & Casadevall, 2012; Grant Steen, Casadevall & Fang, 2013), the effectiveness of scientific gatekeeping (Siler, Lee & Bero, 2015) and the editorial peer-review process (Jefferson, Wagner & Davidoff, 2002). However, although manuscripts submitted for publication undergo critical peer review constituting a fundamental part of the editorial process (but see Enserink, 2001), tools to measure the effectiveness of editors and peer reviewers are rarely provided (Siler, Lee & Bero, 2015; Margalida & Colomer, 2015). In this sense, although most manuscripts show improvement after peer review, errors may still evade detection (Scott Armstrong, 1997).

As a further contribution to the ongoing discussion of how the quality of the peer-review and editing processes can be improved, we recently proposed the application of a standardized mistake index (Margalida & Colomer, 2015). Calculated on the basis of the number of errata published divided by the number of items published, this index could be a surrogate for scientific and editorial quality that will enable temporal trends to be easily evaluated. However, detailed information about the specific mistakes and the analyses of the temporal trends in the effectiveness of the editing process is lacking. To date, we are not aware of any long-term study analyzing these trends or identifying the key errors that occur in papers published after peer review. The approach presented here contributes to improving the editorial and content quality of published papers. Our main goal is to identify the main corrections published and their temporal trends to assess key errors escaping the peer-review and editorial processes. Because three high-quality scientific journals such as *Nature*, *Science* and PNAS can be considered representative of the rigor of published papers and of the effectiveness of peer-reviewing and editing, we undertook a comprehensive analysis of items published by these journals using Web of Science (WoS) during the period 1993–2014. We then applied a more detailed analysis to identify the most common types of mistakes made by authors, which had escaped the peer-review and editorial process. This overview about the trend indicator of quality standards could enable editors, reviewers and authors to reduce the most habitual mistakes and thus improve the scientific quality of published papers.

## Materials and Methods

The database used for this study was compiled from a search of all articles indexed by WoS between 1993 and 2014 published in *Nature*, *Science* and PNAS. According to the WoS classification, we obtained the total number of items in the following categories: *Total*, as the total items (articles and other material indexed in the WoS, mainly published in *Nature* and *Science*, as Editorials, Editors choice, News & Views, News focus, News story, Correspondence, Letters, etc.) published in a year removing the Corrections; *Articles*: as the total papers published in a year including both articles and reviews; *Corrections*: as the total corrections published in a year. With this information, we calculated two different indices. The *Mistake Index Total* (MIT) is the result of the division of the corrections published by the total number of items published in a year. The *Mistake Index Paper* (MIP) is the result of the division of the corrections published by the total number of papers (articles category) published in a year. We expressed this index in percentage to facilitate comparison with other values. Because the *Correction* category in WoS includes all items published with mistakes, not only articles, we applied a correction factor taking into account the average proportion of corrections affecting papers. Based on the mistakes sample analyzed annually (see below), this factor of correction applied to the total number of mistakes was 70.15% for *Nature*, 39.54% for *Science* and 97.72% for PNAS. This is because *Science* and *Nature* publish many more non-scientific items (i.e., Letters, Correspondence, News) than PNAS, which publishes mainly articles and reviews (see proportions of articles with respect to the total number of items in Table 1).

**Table 1:** Application of the mistake index (MIT and MIP) on scientific journals from different disciplines (data obtained from 2014 in WoS) ordered from higher to lower MIT. Note that in MIP no factor correction was applied.

Journal (impact factor)	Items	Papers	Corrections	MIT	MIP
*Cell* (33.116)	632	569	32	5.3	5.6
*Ecology Letters* (13.042)	171	166	5	3.0	3.0
*Trends in Genetics* (11.597)	69	57	2	2.9	3.5
*Nature* (42.351)	4,794	1,629	105	2.2	6.4
PLOS ONE (3.534)	62,868	61,154	1,427	2.2	2.3
*Proceedings of the Royal Society B-Biological Sciences* (5.29)	655	606	11	1.7	1.8
*Water Research* (5.323)	692	681	10	1.5	1.5
PNAS (9.809)	7,909	7,131	104	1.3	1.4
*Blood* (9.775)	6,630	1,281	82	1.2	6.4
*Science* (31.20)	4,486	1,369	49	1.1	3.6
*Scientific Reports* (5.078)	8,020	7,937	78	0.9	0.9
*Trends in Ecology and Evolution* (15.353)	109	82	1	0.9	1.2
*Nature Communications* (10.742)	5,666	5,621	36	0.6	0.6
*Annual Review of Genetics* (18.115)	25	25	0	0	0
*Biotechnology Advances* (3.941)	117	113	0	0	0
*The Journal of Clinical Investigation* (13.765)	563	557	0	0	0

We also calculated the MIT and MIP indexes on some high-quality journals from different disciplines, including some open access journals to assess whether the index was related to impact factor (according to the Science Citation Index in 2014) or the number of items published. For this purpose, we selected some leading journals (impact factor > 3.5) representative of different research areas (multidisciplinary, biomedicine, ecology, chemist, genetics, biotechnology), including a range of impact factors, and a range of number of items published, including some open access journals.

To assess performance in the correction process, for each journal we analyzed 30 corrections/year selected at random totalling 1,980 mistakes (660 per journal). We considered 30 corrections/year as representative for statistical analyses, taking into account that this figure represents on average of approximately 47% of the corrections published in *Nature*, 34% in *Science* and 28% in PNAS during the study period. We analyzed the time-to-correction, defined as the interval in days between the publication of the original item with respect to the date of publication of the correction and where the error had occurred.

To identify the severity of the mistakes and where they occurred, we also defined a qualitative variable only considering mistakes that appeared in articles (*n* = 1,428). Thus, in the analyses we do not include corrections related to retractions. The corrections analyzed are material outside of misconduct and retraction issues. We considered the mistake of low importance (*mild*) when it was a typographical or minor grammatical error (i.e., typos, or mistakes in the title, address, email address, footnote, author name, or acknowledgements); *moderate* when it affected the paper to some degree but not substantially, impacting sentences, figures, results, or references (i.e., mistakes in values, equations, figure axes, table and figure legends); and *severe* when the mistake was substantial such as important modifications in figures, results or conclusions, implying major changes (i.e., figure substantial modifications, re-interpretation of the results, clarification of the conclusions). We also noted when these corrections occurred as a consequence of editorial or printer error (i.e., after the author galley proof correction, the mistake occurred during the printing process).

### Data analysis

Statistical tests were performed in R2.15.2 (http://www.r-project.org). To assess the temporal trend in the different indices or mistake trends we applied the Spearman rank coefficient. Inter-group differences were tested using the analysis of variance (ANOVA) and the Chi-square test to compare absolute values among journals and mistake categories. To address, in part, the problem of testing for a temporal change in the index trend, we applied the Cusum test, which is based on cumulative sums of the values registered over time such that it accounts for small variations. If the variations are not random, those cumulative sums reach a value showing that the differences are statistically significant. Thus, the decision-making is based on the history of the process, and the variable graph at each time *t* is ${S}_{t}={\mathop{\sum }\nolimits }_{i=1}^{t}({\bar {x}}_{t}-\mu )$, with *μ* being the reference value. If the process does not change compared to the reference value, *S*_*t*_ then takes on values around 0. If the process has increased in value compared to the reference the graph will show an increasing trend, and in the opposite case, if the trend of the graph decreases, the reference value decreases. In the case that the process actually changes, these changes will accumulate in the variable *S*_*t*_ reaching a point at which they will be statistically significant. When we need to detect a positive displacement, the variable is plotted as ${S}_{t}={\mathop{\sum }\nolimits }_{i=1}^{t}({\bar {x}}_{t}-\mu -k)$, and *k* is a value related to the displacement to be detected, so that if the trend in the graph is decreasing then this indicates that no changes have occurred. In some cases, as a consequence of randomness of the variables, some points do not follow the declining trend. It is accepted that this growing trend is significant if the increase between the minimum value and the current value represented is three times the displacement to be detected, i.e., 5*k* (decision interval). Similar to the abovementioned methods are graphics to detect negative offsets to represent the variable ${S}_{t}={\mathop{\sum }\nolimits }_{i=1}^{t}({\bar {x}}_{t}-\mu +k)$. If the process does not undergo variation, the graph’s trend is increasing. When the difference between the represented value and the greatest the above has decreased 5*k* we accept that the reference value has decreased. The values *k* and the decision interval were chosen using the average run length (ARL). We estimated the value of the index during 1993–2014 and used the Cusum test to check whether the value changed (i.e., an increase in the upper 12% with respect to the average value obtained during 1993–1997). In this work, the *k* value is half of the displacement to be detected. This involves working with a significance level of *α* = 0.002.

## Results

### Temporal trends

According to the WoS, in the period 1993–2014 the average percentage of corrections published in *Nature*, *Science* and PNAS was 1.57 ± 0.37%, 2.02 ± 0.44% and 1.45 ± 0.31%, respectively. The average *mistake index total* (MIT) and *mistake index papers* (MIP) obtained respectively during this period were: *Nature* (1.4% and 3.8%), *Science* (1.9% and 2.4%) and PNAS (1.2% and 1.3%) with differences among journals in both indices (MIT: *F*_2,63_ = 70.68, *p* < 0.0001; MIP: *F*_2,63_ = 154.69, *p* < 0.0001). When we take the average values obtained during the last five years (2010–2014), the MIT and MIP values were respectively: *Nature* (1.8% and 4.6%), *Science* (2.1% and 2.7%) and PNAS (1.4% and 1.5%).

**Figure 1:**
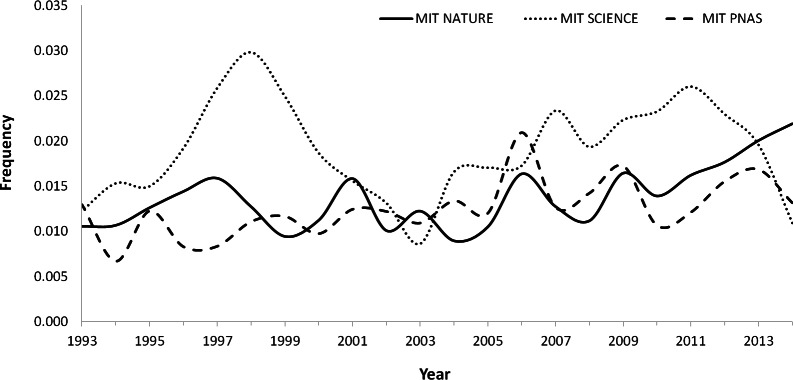
Variation trend in the MIT (*Mistake Index Total;* the result of the division of the corrections published by the total number of items published in a year) in *Nature*, *Science* and PNAS between 1993–2014.

The average annual trend in the MIT during this period increased in *Nature* (0.04%) and PNAS (0.03%), with the increase being statistically significant in both journals (*Nature: r*_*s*_ = 0.56, *p* = 0.008; PNAS: *r*_*s*_ = 0.57, *p* = 0.006). On the contrary, *Science* showed a cyclical pattern with no statistically significant trend (*r*_*s*_ = 0.21, *p* = 0.35) (Fig. 1).

**Figure 2:**
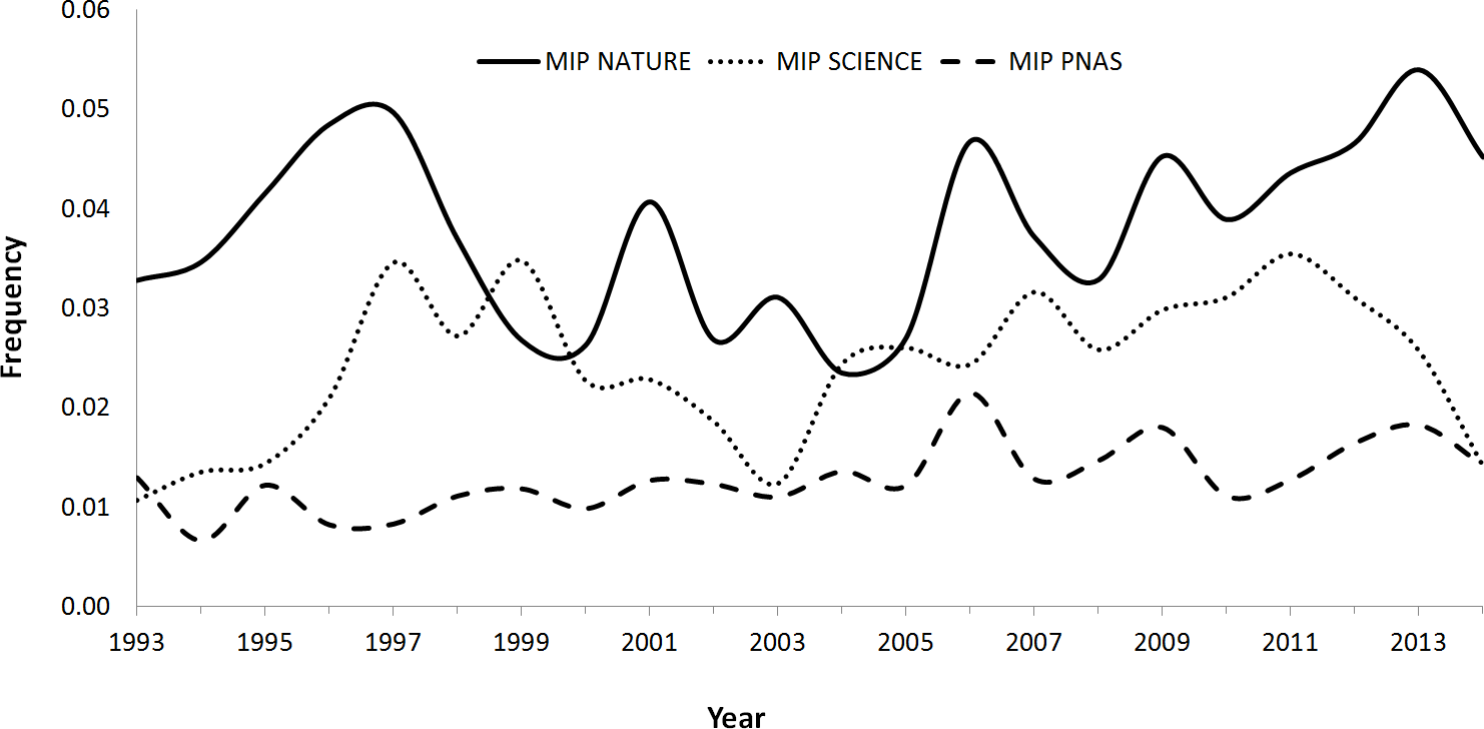
Variation trend in the MIP (*Mistake Index Paper;* the result of the division of the corrections published by the total number of papers published in a year) in *Nature*, *Science* and PNAS between 1993 and 2014.

Because the proportion of articles with respect to the total items published varies among journals (most of the items published in PNAS are research articles, while *Science* and *Nature* publish an important proportion of material considered non-articles), we applied a correction factor (see Methods) to assess the trend in the MIP (i.e., taking into account the proportion of corrections only related to papers). In PNAS, the average annual increase in the MIP during this period was 0.03%, with the trend being statistically significant (*r*_*s*_ = 0.64, *p* = 0.002). On the contrary, no significant trends were observed in *Nature* (*r*_*s*_ = 0.30, *p* = 0.18) or *Science* (*r*_*s*_ = 0.39, *p* = 0.07) (Fig. 2).

**Figure 3:**
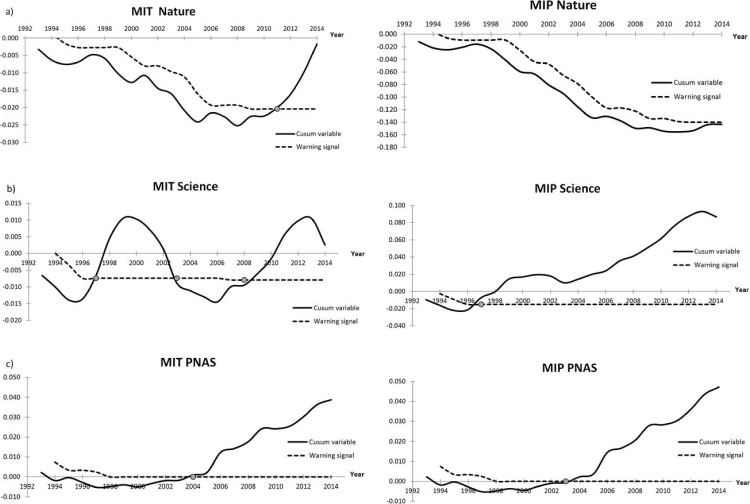
Cusum graphic of the changes in the MIT (left column) and MIP (right column) in *Nature* (A), *Science* (B) and PNAS (C). The graphic was designed to detect changes of 12% with respect to the values obtained from 1993 to 1997. When the warning signal (dashed line) appears below the Cusum variable (line), a significant increase in the index is indicated and is marked with a circle.

The Cusum test showed that, with respect to MIT, there was a shift in the trend in 2008 in *Nature* that was statistically significant from 2011 (Fig. 3). *Science* showed an irregular pattern with periods of significant increase between 1997–2003 and 2007–2014. Finally, PNAS increased its MIT from 2000, with the differences being significant from 2004 to 2014. When we analyzed the MIP (Fig. 3), *Nature* showed increases from 2010, but up to 2014 this trend was not significant. *Science* showed a significant increase from 1995 to 2014. Finally, PNAS showed an increase from 2000 that achieves significance from 2003 to 2014.

### Mistake index and journal impact factor

The application of MIT and MIP on 16 leading scientific journals from different disciplines showed the absence of any relationship to journal impact factor (*r*_*s*_ = 0.123, *p* = 0.651) or the number of items published (*r*_*s*_ = 0.09, *p* = 0.739, Table 1). For example, using the MIT criteria to compare *Nature* and *PLOS ONE* in 2014, we found the same index (2.2) in the two different journals with respect to the number of items published (4,794 vs 62,868), their impact factor (42.351 vs 3.534), the proportion of papers published with respect to the total items (34% vs 97%) and the Open Access policy of the journal (applied only in all papers by *PLOS ONE*).

**Figure 4:**
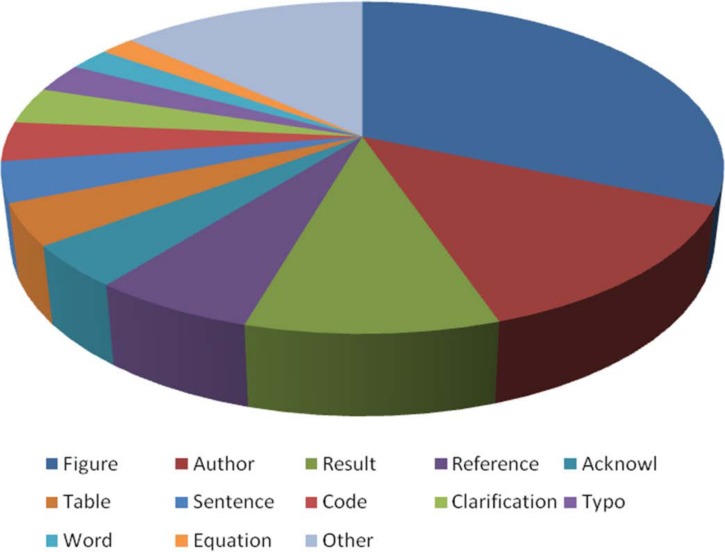
Main mistakes corrected in *Nature*, *Science* and PNAS.

**Figure 5:**
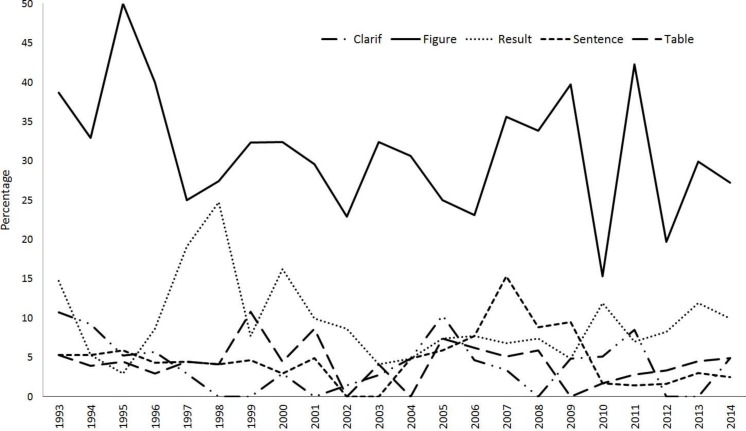
Annual variation in the trend of the main severe mistakes identified in *Nature*, *Science* and PNAS.

### Mistake location, category and editorial responsibility

Of the 1,980 mistakes identified (660 per journal), we only analyzed mistakes related to articles (*n* = 1,428). Of these, 60% of mistakes were related to figures, authors, references or results (Fig. 4). According to the three categories, 47.7% were considered *moderate*, 34.7% *mild* and 17.6% *severe*. When we compared the main types of mistakes in the severe category (Fig. 5), we found similar trends without significant differences (*p* > 0.1 in all cases).

**Figure 6:**
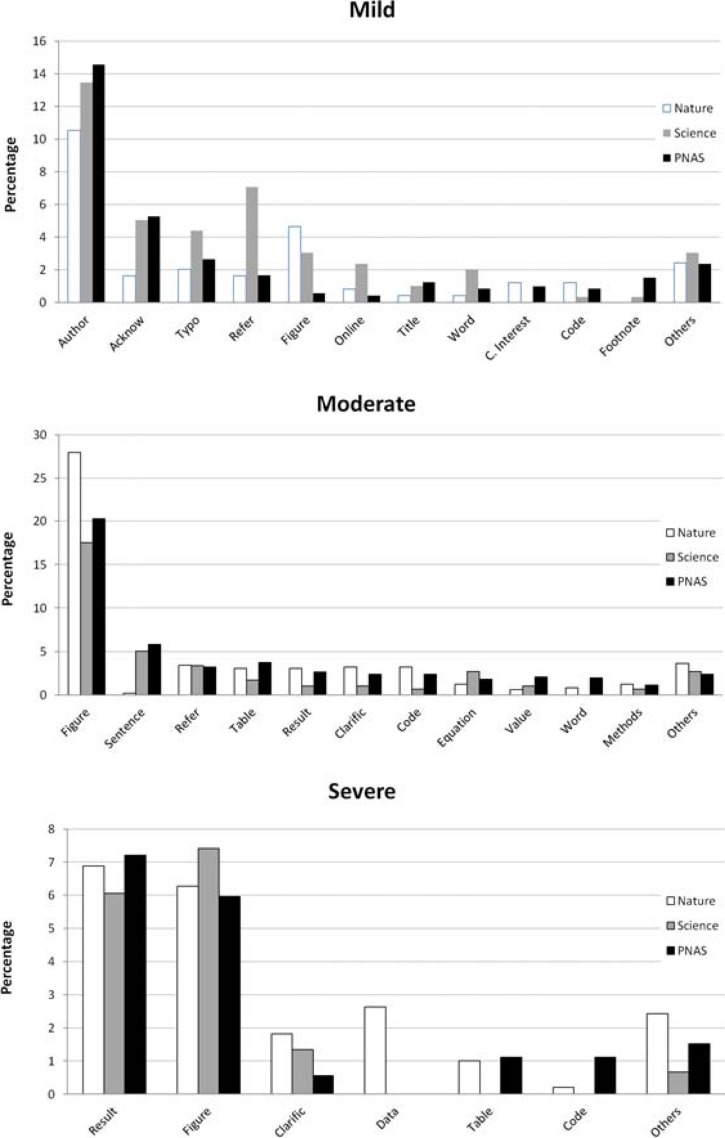
Different types of mistakes according to the category and journal.

When we analyzed mistakes taking into account the three category levels (*mild*, *moderate* and *severe*, see Methods), we found significant differences among journals (${\chi }_{4}^{2}=31.07$, *p* < 0.0001, Fig. 6). Mistakes in *Science* were dominantly *mild* (47.5% vs 28.3% in *Nature* and 36% in PNAS) with the proportion of *moderate* mistakes lower in *Science* (37.9%) than in *Nature* (51.4%) and PNAS (49.7%) and non-significant differences among the proportions of *severe* mistakes (*Nature* 20.3%, *Science* 14.6% and PNAS 14.3%).

The printing process was responsible for 5% of mistakes in *Nature*, 2.7% in *Science* and 18.4% in PNAS, with the differences being statistically significant (${\chi }_{2}^{2}=117.73$, *p* < 0.0001).

### Time-to-correction

**Figure 7:**
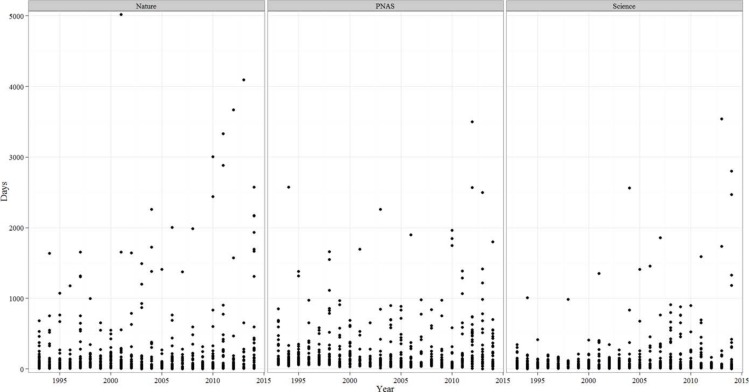
Annual variation in the time-to-correction in *Nature*, PNAS and *Science*.

The time interval between publication and correction varied significantly among journals, with the shortest time-to-correction in *Science* (*Nature*: 232.77 ± 693.16 d; *Science*: 136.50 ± 304.20 d; PNAS: 232.09 ± 356.86 d; *F*_2,2,107_ = 9.185, *p* < 0.0001). When we analyzed the trend in the time-to-correction (Fig. 7), in all journals we found an increasing trend that was statistically (or marginally) significant (*Nature r*_*s*_ = − 0.081, *p* = 0.032; *Science r*_*s*_ = 0.103, *p* = 0.07; PNAS *r*_*s*_ = − 0.096, *p* = 0.009). When we only considered the time-to-correction in articles, the trend was also statistically significant (*Nature r*_*s*_ = 0.109, *p* = 0.016; *Science r*_*s*_ = 0.119, *p* = 0.041; PNAS *r*_*s*_ = − 0.098, *p* = 0.008).

For the three mistake level categories, we found significant differences in time-to-correction with corrections of *severe* mistakes taking longer (*mild*: 185.89 ± 704.85 d; *moderate*: 198.97 ± 320.17 d; *severe*: 467.96 ± 602.46 d, *F*_2,1,493_ = 30.3, *p* < 0.0001). In all three journals, *severe* mistakes implied significantly (*p* < 0.0001) longer correction periods.

## Discussion

The index outlined here is a simple method for assessing peer-review and editorial quality by providing a tool for editors. The application of the mistake index provides a new perspective on the quality of the peer-review and editing processes, allowing editors to easily measure the effectiveness of the peer-review and editorial process over time.

Our findings suggest that the trend in the number of mistakes published in *Nature*, *Science* and PNAS, although still relatively small, is increasing progressively. This pattern coincides with the increasing rates on retracted articles among top journals (Feng Lu et al., 2013). Regardless of the mistakes severity, from an editorial point of view, this can be interpreted as (1) a decreasing trend in the quality of peer-review and editorial processes in these journals, or (2) an increase in the trend detection and publication of correction notes. In this sense, the temporal pattern is important to take into account because it may also be argued that being willing to publish corrections is an indicator of quality of editorial practice, and that a low number of corrections might indicate an unwillingness to acknowledge errors.

According to our results, the Cusum test applied to both indices suggests different temporal patterns. For MIT, the significant increase takes place during the last decade, whereas for MIP the significant shift took place 18 years ago in *Science* and 12 in PNAS, with and no significant changes in *Nature*, although the trend forecasts a significant increase over the next few years. Although we do not evaluate the trends in other journals, the comparative values of both mistake indices obtained from other scientific disciplines (Table 1) suggest a similar proportion of corrections published.

In regards to the type of corrections, 17.6% were considered *severe*. Thus, these findings suggest that this is not a negligible problem and that the peer-review and editorial process can be improved by identifying the most common mistakes. We found that figures are involved in 32% of the corrections identified, and should be a priority during the peer-review and editorial process, in an effort to reduce the publication of corrections. This category alone encompasses 41.3% of *severe* mistakes and 49.3% of *moderate* mistakes. Given the relationship of figures to the results of a paper, for the *severe* category, figures and results account for 79.4% of the mistakes identified. Thus, providing guidelines to reviewers, authors and editors that focus on these areas is recommended to reduce mistakes and improve the quality of published manuscripts.

With respect to the time taken to publish a correction, the trend in the time-to-correction increased significantly over time in *Nature*, *Science* and PNAS. This seems a contradictory pattern taking into account that in a digital era with a more advanced technologies and editorial tools, the time-to-correction was more efficient during 90’s that currently. A possible explanation of this unexpected result could be the initial reluctance of some authors and/or editors to publish corrections. Since the prestige of the author and the journal can be affected with the publication of mistakes identified, one possible action is the delay in the decision to inform (author) or to publish (editor) the corrigendum. Analyzing the three journals, on average *Science* was the fastest journal in publishing corrections. However, in all three journals, the publication of *severe* mistakes took place significantly later that in the other two categories. *Severe* mistakes were published 1.23 years after the publication of the original paper. In contrast, Corrigendum for *mild* and *moderate* mistakes were published 0.51 and 0.54 years after the publication of original papers, respectively. A possible explanation could be related to the complexity of the mistake. *Mild* and *moderate* mistakes are probably easier to identify and correct in a timely fashion. In contrast, *severe* mistakes are more difficult to identify and the assessment required to confirm the mistake is a more elaborate process.

In various disciplines, decisions made by medical professionals, economists, engineers, managers and policy-makers are (or should be, see Margalida, Kuiken & Green, 2015) based on available evidence and, long delays in the correction process can exacerbate the dissemination and implementation of incorrect information. Thus, it is necessary to improve efforts to detect and reduce the time of correction of *severe* mistakes. In this regard, editors and authors should be encouraged to publish their corrections as soon as possible.

It is difficult to disentangle the factors affecting the increasing trend in published mistakes. However, we can speculate about several factors that may be contributing to this. The pressure to publish reduces the time invested by authors to carefully revise their manuscripts, and impacts the reviewing process. The quality of the scientific literature depends on time dedicated by conscientious reviewers and sometimes good reviewers are less likely to review manuscripts and/or the time invested in the review is insufficient as a consequence of shorter deadlines and editorial process (in this case with the pressure to publish as soon as possible). According to several studies, on average, reviewers spend between two and six hours in reviewing a paper (Jauch & Wall, 1989; King, McDonald & Roderer, 1981; Lock & Smith, 1990; Yankauer, 1990). Although *severe* mistakes make up a relatively small proportion (between 14 and 20%) of errors, moderate mistakes range between 38 and 51% and authors and editors should focus on their detection. On the other hand, it is important to note that 13% of the mistakes analyzed were related to the author category (misspelling, missing co-author, incorrect address). These are “minor” corrections, but indicate a superficial review of the accepted manuscripts, in this case being the responsibility of the authors and the editorial process.

Future research could explore the types of papers more typically involved in these mistakes by field and discipline, the spatial scale of errors based on the country of origin of lead authors and if the Open Access factor could have some influence.

Peer review is so well established that it has become part of the system for assessing academic merit in appointments and promotions (Jefferson et al., 2002; Bornmann, 2013). Given that peer reviews are an essential step in the editing process, and that reviewers work for no extrinsic rewards with time constraints considered a major handicap, journals must seek appropriate alternatives that will the stimulate the recruitment of good reviewers and assure the maintenance of quality standards in published manuscripts. As such, the next challenge is to seek some form of effective incentives (Hauser & Fehr, 2007) for reviewers such as, for example, the establishment of a recognized reviewer quality index (Paoletti, 2009; Cantor & Geo, 2015). This may increase the recruitment of reviewers to assist with the peer-review and editorial processes and provide some return on investment for the altruistic work carried out by reviewers. Finally, many manuscripts are not reviewed by the best in the field, since these experts are frequently overwhelmed and many conserve their time for their own research (Liesegang, 2010). Thus, we need to change the paradigm and assume that publication should be the start of the reader peer-review process (Horton, 2002; Liesegang, 2010). Post-peer review should be encouraged by editors and assumed as a part of the process by readers and authors.

## Supplemental Information

10.7717/peerj.1670/supp-1 Data S1 Dataset for Supplementary Information

## Additional Information and Declarations

### Data Availability

The following information was supplied regarding data availability: The database has been added as Data S1.
